# Hydrogen 4.0: A Cyber–Physical System for Renewable Hydrogen Energy Plants

**DOI:** 10.3390/s24103239

**Published:** 2024-05-20

**Authors:** Ali Yavari, Christopher J. Harrison, Saman A. Gorji, Mahnaz Shafiei

**Affiliations:** 1 School of Science, Computing and Engineering Technologies, Swinburne University of Technology, Melbourne, VIC 3122, Australia; cjharrison@swin.edu.au (C.J.H.); mshafiei@swin.edu.au (M.S.); 2 Hydrogen 4.0 Lab, Swinburne University of Technology, Melbourne, VIC 3122, Australia; saman.asgharigorji@deakin.edu.au; 3 6G Research and Innovation Lab, Swinburne University of Technology, Melbourne, VIC 3122, Australia; 4 Department of Aerospace and Aviation, School of Engineering, Royal Melbourne Institute of Technology, Melbourne, VIC 3001, Australia; 5 School of Engineering, Deakin University, Melbourne, VIC 3122, Australia

**Keywords:** hydrogen, Industry 4.0, renewable energy, green hydrogen, Internet of Things, Hydrogen 4.0

## Abstract

The demand for green hydrogen as an energy carrier is projected to exceed 350 million tons per year by 2050, driven by the need for sustainable distribution and storage of energy generated from sources. Despite its potential, hydrogen production currently faces challenges related to cost efficiency, compliance, monitoring, and safety. This work proposes Hydrogen 4.0, a cyber–physical approach that leverages Industry 4.0 technologies—including smart sensing, analytics, and the Internet of Things (IoT)—to address these issues in hydrogen energy plants. Such an approach has the potential to enhance efficiency, safety, and compliance through real-time data analysis, predictive maintenance, and optimised resource allocation, ultimately facilitating the adoption of renewable green hydrogen. The following sections break down conventional hydrogen plants into functional blocks and discusses how Industry 4.0 technologies can be applied to each segment. The components, benefits, and application scenarios of Hydrogen 4.0 are discussed while how digitalisation technologies can contribute to the successful integration of sustainable energy solutions in the global energy sector is also addressed.

## 1. Introduction

Hydrogen is expected to be the energy carrier of the future [1]. The Internation Renewable Energy Agency (IRENA) estimates that a global electrolyser capacity of 5.7 GW producing approximately 523 million tons of hydrogen per year will be required to limit global warming to 1.5 °C [2]. This is many multiples of the current production capacity, and to reach this target in the next 26 years is a grand challenge. Today, the majority of the hydrogen is produced by either reforming natural and/or refinery gas or by Coal Gasification (CG). Together these methods account for 66% of global hydrogen production [3]. Although electrolysis using green energy sources has clear benefits in respect to the environment and global warming, hydrogen production using electrolysis contributes to only 4% of global hydrogen production [3]. Achieving the IRENA goal of 97% [1] will involve concentrated effort to lower the cost of construction and the operation of green hydrogen plants.

The key challenges for the use of electrolysis in producing green hydrogen are as follows: (i) electricity, efficiency, and equipment costs when compared with established fossil-fuel-based approaches; (ii) complexity of compliance monitoring including traceability of “green” products; and (iii) operation and preservation of plant equipment and safety of personnel. Advancements in innovative technologies—such as the emergence of smart sensing, analytics, and advanced control algorithms—have improved the efficiency of production, accuracy, and timeliness of monitoring; improved compliance with standards and regulation; and increased safety across a broad spectrum of industries, particularly in the manufacturing sector. The term “Industry 4.0” has been coined to describe this intersection of innovative technologies and generally represent the application of emerging digital technologies to industrial settings.

Hydrogen production plants have the potential to gain similar operational and safety advantages from implementation of advanced data analytics, use of Internet of Things (IoT) devices and infrastructure, and digital innovative technologies such as Digital Twins (DTs) to improve efficiency and reliability, particularly for systems that integrate electrolysis into the green energy mix. These advantages also provide avenues for greater assurance on the provenance of hydrogen generated from renewable sources. Other advantages include real-time monitoring and maintenance of plant equipment, increased personnel safety, and real-time compliance assurance. Incorporating this Industry 4.0 style paradigm into systems for hydrogen production, storage, distribution, and use is, in essence, the philosophy inherent in the next generation of energy systems that we term “Hydrogen 4.0”.

In particular, we define Hydrogen 4.0 as the integration of hydrogen production, storage, distribution, and utilisation systems with digitalisation capabilities of the Fourth Industrial Revolution (i.e., Industry 4.0), such as IoT, DT, Artificial Intelligence (AI), and advanced data analytics’, enabling a green and renewable energy transition.

The main aim of this paper is to investigate how hydrogen power plants can benefit from interoperability and the enhanced efficiency gained through the Hydrogen 4.0 paradigm.

The rest of this paper is organised into the following sections: Section 2 will summarise the current literature relevant to hydrogen and Industry 4.0 concepts. Section 3 will provide an overview of hydrogen plants in respect to conversion of electrical energy to and from hydrogen gas and the technologies involved with internal energy management (e.g., micro-grids) and hydrogen production. Further, this section discusses the distinctive features of renewable and non-renewable hydrogen plants. Section 4 will present the Hydrogen 4.0 paradigm and discuss how it is enabled by emerging cyber–physical systems. Section 5 will present a number of example scenarios that benefit from the Hydrogen 4.0 paradigm. Finally, Section 6 concludes this paper and discusses the future directions.

## 2. Related Works

In this section, we review relevant studies on the description and development of Industry 4.0 and DT technologies in the energy sector.

DTs form an important component of the Industry 4.0 paradigm, enabling computational modelling and performance analysis of complex plant equipment without involving real-world equipment. A review on the application of DTs in the energy sector has been conducted with a focus on three different perspectives including production, storage, and consumption [4]. This work does not focus on a specific energy source per se but considers DT applications in any energy generation sector, including thermal, renewable, fossil fuel, and nuclear. Ghenai et al. [4] showed that only 5% of publications on the application of DTs in the energy sector focus on use of hydrogen. In addition, they reported that 63.75% of the publications on DT applications in the energy sector focus on energy consumption, 22.5% focus on energy storage, and only 13.75% focus on energy production.

A detailed technical review [5] focuses on the application of DTs in the electrochemical, mechanical, thermal and chemical sectors of the energy industry. This review notes that DTs provide a potential means to enhancing performance, lowering maintenance and operational costs, and ensuring safe operations in renewable energy systems that include energy storage. This work discusses the DTs in energy sectors from four different perspectives including context, life cycle, function, and architecture and argues that due to the complexity of different energy sector characteristics, the DT in each sector requires a unique architecture to fully address the requirements of the related sector.

Sleiti et al. [6] focused on architecture aspect of DT application in energy production sector. They argue that application of DT in power plants is a complex task which requires a comprehensive and robust architecture design, and such comprehensive DT architecture in energy production sector for power plants is missing. Therefore, they conducted a review in this area and subsequently proposed a new architecture, which they reported achieved high reliability, availability, and maintainability at lower cost.

Singh et al. [7] conducted a review regarding the application of digital and physical twins across different industries including the energy sector. They indicated that DT enables an increase in the efficiency of power production, improvement of the predictive maintenance by predicting the components of the life cycle and potential future failures, and an increase in the system reliability by enabling real-time data collection, simulation, and analysis. Such characteristics of DT can significantly improve the decision-making process, which in the energy sector, can result in a substantial amount of financial gain.

Yu et al. [8] conducted a systematic review on energy DT for industrial energy management and categorised the drivers underpinning the research in energy DTs, which included profit (18%), decolonisation (27%), safety and risk (5%), quality (4%), throughput (14%), and energy efficiency (32%). They argue that the share of publications in energy DT is significantly low, with around 50 papers, compared to that of building DT, with 400 plus, and manufacturing DT, with more than 1000 publications. This indicates that the role of DT in improving the energy sector output is undermined and that there is significant room for potential research to be conducted in this area.

Errandonea et al. [9] conducted a review on the role of DT on maintenance across different industries. Their review showed that after the manufacturing sector, the energy industry has the second highest number of publications regarding DT application in the maintenance process. They categorised the application of DT based on different types of maintenance strategies including (1) reactive maintenance, (2) preventive maintenance, (3) condition-based maintenance, (4) predictive maintenance, and (5) prescriptive maintenance. They argue that DT revolutionises the predictive and prescriptive maintenance strategies by enabling integration of real-time and historical data to identify the potential failure points in the system. Therefore, a set of actions can be formulated to be regularly performed at a certain time interval to prevent any future failure in the system. This review shows that the predictive maintenance enabled by DTs is by far the most investigated maintenance strategy in the energy sector in the existing literature.

He and Bai [10] focused on reviewing the existing literature that address the role of DT in streamlining the manufacturing process and achieve a sustainable manufacturing environment. They discuss how DT can help to optimise the manufacturing process from different perspectives, including product design and manufacturing, logistics planning, manufacturing process optimisation, and sales. Such an approach can help to identify the shortcomings in existing processes throughout a product life cycle and create a macro view regarding the impact of these shortcoming on different aspect of the manufacturing life cycle. This can potentially enable the decision makers to increase their process optimisation and improve manufacturing sustainability. This kind of end-to-end DT application in hydrogen energy production is missing in the existing literature, which is a driving motivation for conducting this review.

Existing related works on DTs reveal a research gap in the intersection of Industry 4.0 and hydrogen-enabled power plants. While works such as [5,6] discuss hydrogen energy, they primarily focus on storage [5] or DT architecture [6] rather than on hydrogen power plants. Other reviews [7,8,9,10] explore DTs within the broader energy sector but fail to address hydrogen-specific applications. Hydrogen energy production, characterised by its zero-emission output and high energy per mass, fundamentally differs from traditional energy systems and requires specialised discussion from a digital twin perspective. Its storage and conversion technologies, demanding precise monitoring and control systems, are ideally suited for optimisation through DT technologies. This requires unique DT considerations, such as the integration of advanced sensor data and predictive analytics, to manage hydrogen’s volatile nature effectively. Such tailored approaches are crucial for mitigating risks and enhancing efficiency in hydrogen systems, underscoring the need for dedicated DT research in this emerging field. Our review addresses these gaps by providing an end-to-end analysis of DTs in the hydrogen sector, covering production, maintenance, sensors, storage, processes, and distribution to end-users. This comprehensive evaluation of DT impacts across the hydrogen life cycle offers significant insights into improving the sustainability of hydrogen energy production and utilisation from economic, social, and environmental perspectives.

## 3. Hydrogen Plant Overview

A hydrogen plant is a facility that consumes or generates electrical energy to produce hydrogen (H_2_) gas. This may optionally include infrastructure to import, store, and/or export electrical energy to or from the grid or co-located facilities. Furthermore, such plants may consider the H_2_ energy carrier as an import or export product or as a local storage medium akin to a battery. Hydrogen produced exclusively using renewable energy sources is commonly labelled as “green” hydrogen even though it is chemically and visually identical to hydrogen produced via any other production method. These plants may employ one or more power-to-gas conversion (e.g., conversion of electricity to H_2_ via electrolysis) and gas-to-power (e.g., conversion of H_2_ to electricity via fuel cells) processes [3,11] thereby creating a closed-loop process referred to as power-to-gas-to-power.

**Figure 1:**
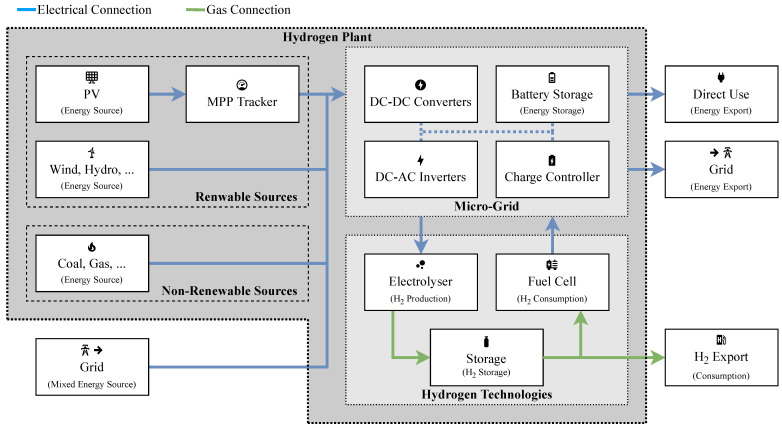
Simplified block diagram of a hydrogen production plant integrating energy storage, hydrogen production and consumption, hydrogen storage, and several avenues for potential energy or hydrogen import/export.

A simplified block diagram of a general-purpose hydrogen plant is illustrated in Figure 1. This illustrates how the major components of such plants are interconnected and provides an overview of how electrical energy and hydrogen is routed between components. The major constituent components are outlined in the following subsections, including electric energy production and storage; hydrogen production, storage, and consumption; and plant imports and exports.

### 3.1. Energy Sources and Storage

The classical definition is energy is a means to do work. It is available in a variety of forms (electrical, thermal, kinetic, nuclear, etc.) and can be tapped from a variety of sources. In hydrogen plants, H_2_ is typically not involved in the generation of energy; instead, it is an energy carrier used as a means to transport and store energy. Traditionally, many sources of energy tapped by humidity to provide electric power have been “non-renewable”, the most significant sources being coal, oil (petrochemicals), and natural gas [12]. This is rapidly changing, as “renewable” energy systems are emerging as economic means of generating energy at the national and international scale, including sources such as solar (Photovoltaic (PV) panels), wind, geothermal, and hydraulic (hydroelectric) [13].

Energy storage media—such as batteries—are not in and of themselves renewable or non-renewable and bring forth an argument as to the true meaning of these terms when an energy system may handle renewable energy but os constructed using non-renewable resources and materials. This matter is a complex debate beyond the scope of this work, which will classify only the provenance of energy in terms of renewability. In different scenarios, multiple energy sources may be combined to shape an energy system at scale, such as a power grid, to supply a load or an end-user [14]. In a hydrogen plant, energy sources can include on-site resources, co-located facilities with dedicated interconnects, and remote or grid-connected sources.

### 3.2. Micro-Grid

This section details the essential infrastructure for electric energy management within hydrogen plants, including its vital role in connecting to external power networks. It emphasises the importance of diverse components that regulate and route Alternating Current (AC) [15] and Direct Current (DC) [16] power according to the operational needs of plant equipment. This regulation is achieved through a variety of rectifiers, DC-DC converters, and inverters, strategically organised around a central AC or DC bus. The section further explores how the micro-grid handles instability and inconsistency in energy sources through short-term solutions such as capacitors and long-term strategies such as battery storage. The choice of components within this framework is driven by the power handling and energy storage capacities required for the micro-grid’s effective operation. Additionally, it highlights the necessity of devices for electrical the isolation of components during faults or maintenance periods [17].

#### 3.2.1. AC Components

AC components, particularly inverters, are necessary to convert the DC power output from PV panels, battery storage, fuel-cells, or other sources to AC power of a specific frequency and amplitude (dictated by local standards and regulations). AC power is widely accepted by commercial hydrogen production equipment or supplementary consumers within the plant or within adjacent facilities and can be supplied directly to the mains grid as an export. As with all energy conversion, an efficiency cost is associated with this process that can be reduced through the selection of appropriately rated components [18]. Typical residential or commercial solar cell installations may aggregate a number of separate PVs into an array before combining that power in a single large inverter. Increasingly, PVs are supplied from large manufacturers with pre-installed micro-inverters. These micro-inverters perform the same role as their larger brethren but overcome some of the efficiency challenges faced in small-to -mid-scale installations, particularly where occlusion of one or more panels in a string may occur.

As AC power, electrical energy can be used with most commercially available hydrogen production equipment, used immediately in consumer or industrial equipment, or supplied to the grid. As with all energy conversion, an efficiency cost comes with this process that can be reduced through proper power rating. A typical residential or commercial solar cell installation may aggregate a number of separate PVs into an array before combining that power in a single large inverter. Increasingly, PVs are supplied from large manufacturers with inverters pre-installed. So called micro-inverters perform the same role as their larger brethren but overcome some of the challenges faced in small to mid-scale installations. Arrays of PVs tend to operate at the Maximum Power Point (MPP) of the least performant cell in the array. If one cell in the string is obscured from direct illumination, subject to widely variable operating conditions (for example localised heating), or outright fails, then the performance of the entire array is degraded. Micro-inverters circumvent this issue by sourcing power from each cell individually before aggregating that power in the AC portion of the micro-grid (or mains grid in the case of residential installations).

Whilst well-suited to the variable installation conditions found in residential and small commercial settings, the use of micro-inverters is not without its drawbacks. Use of multiple discrete inverters increases cost, decreases overall efficiency, and increases the scope for failure of the individual components. It is additionally worth noting that many residential inverters are not suitable for use in a micro-grid as they must conform to UL 1741 [19]. Such inverters are designed to be disciplined by the mains grid and match their output only when such a supply is available—an important safety feature that prevents back-driving of power lines in the event of power outage or during line maintenance. In mid-to-large-scale energy production, the use of monolithic inverters are often preferred, as it offers economies of scale and greater flexibility over those used in small-scale installations.

#### 3.2.2. DC Components

The discussion on DC components centres on the functionality of PV panels and the critical role of Maximum Power Point Tracker (MPPT) controllers [20]. PV panels are semi-conductor devices with complex current-voltage characteristics. Ultimately, this means the output characteristics of a PV panel depend upon the load conditions, with a peak efficiency occurring somewhere between Open Circuit (OC) and Short Circuit (SC) conditions, called the MPP. MPPT controllers are typically integrated with DC-DC converters to monitor for and drive load conditions towards operation at the MPP. Thus, proper engineering of any DC-DC converter must take into account the range of input and output conditions and provide good conversion efficiency across this range. MPPT controllers typically feed directly into battery storage, another essential component in any renewable energy system. Battery storage is necessary to offset the volatility associated with many renewable energy sources, namely solar and wind generation. While the total energy storage capacity of batteries is ultimately limited by cell chemistry and size, the ability to handle rapid changes in load or charge conditions make them ideal for mid-to-long-term energy storage. DC-DC conversion can be accomplished through a variety of topologies, the selection of which depends upon the desired change in voltage or current characteristics. Voltage can be increased using boost topologies and decreased using buck topologies. Topologies can be combined to accommodate variable inputs and outputs, with the operating modes being switched as required, and include buck-boost and Single-Ended Primary-Inductor Converter (SEPIC). A number of more esoteric topologies exist for specific applications but are beyond the scope of this work.

### 3.3. Hydrogen Production

The various methods of hydrogen production generate H_2_ that, for most intents and purposes, is chemically and physically identical to that produced via any other production method. Although hydrogen is considered to be a clean and non-toxic energy source [21,22], certain production methods produce undesirable by-products (i.e., greenhouse gases such as carbon monoxide (CO) and carbon dioxide (CO_2_)) [23], and thus there is substantial interest in classifying the provenance of H_2_. Although a number of sophisticated formal systems exist for this purpose, a simple “colour” system has emerged as an accessible and widely understood means for classification of H_2_ depending upon production method, feedstock, and by-products despite the primary energy source used for generation being the dominant factor in this system [23,24]. Equipment vendors sometimes create new or hybrid colours to describe their specific process [25], and there are several hydrogen colours that are widely accepted:

- Gray: generated using fossil-fuel feedstock(s) and energy in processes that generate greenhouse gases.
- Blue: generated as described above but with Carbon Capture and Storage (CCS) being used to arrest greenhouse emissions.
- Green: generated using energy derived exclusively from renewable sources.

Process-oriented classification is also used to differentiate production methods. Production processes are segregated according to the specific production mechanism and/or materials used to produce hydrogen, including feedstock. This general classification includes (i) electrochemical methods, (ii) thermochemical methods, and (iii) biological methods [26]. The electrochemical process includes either electrolysis or photolysis [27] and is the most dominant means of green hydrogen production, specifically involving water splitting via renewable energy sources. The thermochemical method includes Partial Oxidation of Methane (POM), CG, and Steam Methane Reforming (SMR) [28], which are among the most established methods for industrial hydrogen production at scale today. Biological methods—as the name implies—rely upon biological processes to generate hydrogen and include fermentation, pyrolysis, etc. One can also consider other methods, such as high-temperature electrolysis, which may be classified as a combination of the aforementioned methods.

As the simplest means of generating green hydrogen, electrolysis forms the basis of renewable hydrogen plants. Electrolysis involves the splitting of H_2_ and Oxygen (O_2_) gas from liquid Water (H_2_O) using electric power. This process is the key to the production of green hydrogen, as it enables the use of energy from renewable sources to be used in the production of hydrogen directly. This approach remains expensive when compared to some of the more well-established methods of hydrogen production that rely upon carbon-based feedstocks or energy from carbon-emitting sources. However, recent research has focused upon the cost reduction and longevity of electrolysis equipment, and thus the cost of hydrogen produced in this way has steadily decreased. An additional benefit of the electrolysis approach is the production of oxygen as a secondary product, which can be captured and used in a variety of chemical and bio-chemical processes.

Various technologies can be used to undertake hydrogen production using electrolysis, each with varying degrees of production efficiency, hydrogen purity, life span, scalability, resistance to poisoning or damage by contaminants, and operating costs. In principle, electrolysis requires only an electric current, an electrolyte, and conductive electrodes to break down H_2_O molecules into their constituent components. However, this naïve approach is not particularly efficient and scales poorly. Currently, the most widely available approach for hydrogen production employs Proton Exchange Membranes (PEMs) to optimise production, although a substantial limitation of this technology is the dependency upon expensive noble metal catalysts, including platinum. Additionally, recent advancements in competing technologies using Solid-Oxide Electrolysis (SOEL) and Anion Exchange Membranes (AEMs) have shown these technologies hold significant potential, although some are still far from commercial availability.

### 3.4. Hydrogen Storage

Hydrogen can be stored in a range of different ways. Classically, hydrogen can be stored as a compressed gas or cryogenic liquid or bound to a nano-porous material [29]. Hydrogen storage in the form compressed gas requires high-pressure tanks ranging from 300 to 700 bar, whereas the liquid hydrogen requires lowering down the temperature to a cryogenic temperature (≈−259${\;}^{\circ }C$), with the solid storage mainly occurring via absorption. There are other material-based methods as well (one might still categorise them as the three mentioned categories), in which the hydrogen molecules are bound to a liquid carrier such as ammonia (NH_3_) or toluene (C_7_H_8_) or in the form of a solid carrier such as interstitial hayrides or non-interstitial hayrides. The main challenge in hydrogen storage relates to the portable applications, where a tradeoff needs to be made between the density, size, cost, and safety. This is less of a challenge in stationary applications [30].

## 4. Hydrogen 4.0 Architecture and Implementation

A future economy founded upon the tenants of Industry 4.0 and tightly integrated with hydrogen energy will be built upon systems coupling artificial software models with real-world hardware. The renewable hydrogen plant as an ideal example of a complex power-to-gas and gas-to-power system and signifies the genuine requirement for a cyber–physical system. Renewable energy sources are intermittent and can be unpredictable, making optimisation of plant operation challenging. There is undeniable benefit to such systems when capabilities for real-time monitoring and control, predictive maintenance, advanced analytics, and remote operation are enabled through the addition of Industry 4.0 paradigms to emerging hydrogen industries. Some of the key components and technologies required to achieve this vision are outlined in the following sections.

### 4.1. Hydrogen Sensors

As an odourless, colourless, and tasteless gas with a nearly invisible flame, the detection and quantisation of hydrogen gas without specialised equipment is challenging, yet accurate and reliable detection of hydrogen gas is essential for plant safety, processes monitoring, and quality assurance. Hydrogen represents a significant safety hazard wherever it is used, as many of the chemical properties that make it useful as an energy carrier are also associated with extreme volatility. The ignition energy of hydrogen gas is lower than that of other fuels (0.02 mJ), has a high heat of combustion (142 kJ/g), and is flammable across a broad concentration range (approximately 4 to 75%_vol/vol_ although the exact limits depend upon environmental conditions) [31,32]. The low molecular weight of hydrogen gas, the lowest of any molecule, makes it extremely buoyant and liable to accumulate within enclosed spaces, and as such, even slow leaks represent a substantial safety hazard [33,34]. Many fires and explosions within hydrogen power plants have been reported, and this risk is set only to increase with the widespread deployment of hydrogen-based infrastructure [35]. There is also a substantial need within this infrastructure to accurately quantise high concentrations of hydrogen gas or to identify contaminant compounds within a mixture dominated by hydrogen gas. This measurement is essential to the long service life of components such as fuel-cell and electrolyser membranes that are easily damaged by contaminant compounds [36,37,38].

Traditionally, the detection and quantification of hydrogen gas were performed using analytic processes such as gas chromatography, mass spectrometry, or gas ionisation [39]. These approaches exploit various physical characteristics of hydrogen to perform detection and measurement but typically utilise instrumentation that is physically large, expensive, and costly to operate and requires specialised knowledge to operate and maintain [40]. Such an approach is slow, requiring sample capture, transport to a dedicated facility, time on shared instrumentation, and dedicated personnel to generate and interpret results. Electronic hydrogen gas sensors trade some of the specificity enabled by traditional methods to perform the rapid detection of hydrogen gas concentration in a miniaturised form factor. These devices produce a measurable electronic output that can be correlated to known concentrations of hydrogen either within a process stream or in the immediate environment. In some cases, detection of other compounds can be used for the indirect detection of hydrogen, such as the use of oxygen (O_2_) sensors to detect drops in ambient oxygen associated with the forming of water molecules [41].

Many different types of hydrogen gas sensors have been proposed in the literature, and a number of technologies have advanced to commercial viability [32]. Hydrogen sensors are available in a range of form factors suited to particular applications. These range from handheld devices used by plant workers to monitor for gas hazards during work (particularly in confined spaces) through to fixed installations for process monitoring. Across such a broad range of environments—and with this diversity set only to increase—maintaining accuracy and long-term reliability is challenging, particularly when environmental conditions may be unstable and contaminant compounds present. It is therefore unsurprising that the development of hydrogen sensors has remained an active area of research and forms a key component of the Hydrogen 4.0 paradigm from the perspective of generation, storage, and use. Hydrogen gas sensors rely upon a limited set of physio-chemical interactions to perform sensing, each with associated advantages and drawbacks [32]. Specific sensor performance targets depend heavily upon application, with automotive use presenting perhaps the most demanding use case, owing to the highly variable operating conditions and environments [32,42,43]. Although a range of methods are available for detection and quantisation of hydrogen, commercially available devices tend to focus on those methods that are economic, scalable, and well-established—even if such characteristics come at the cost of sensitivity or selectivity. A range of commercially available hydrogen gas sensors for safety applications are presented in Table 1.

These devices are classified by a range of metrics including sensor format, sensor type, sensor characteristics (range, resolution, and response time), rated operating conditions (temperature and humidity), approximate service life, oxygen requirements (enabling use in aerobic or anaerobic environments), rating for use in explosive atmospheres (compliant with ATEX or IECEx ratings), and relative cost. Sensor format encompasses the typical configurations of the hydrogen gas sensors: as complete units with integrated electronics to generate measurements in either fixed or portable formats or as bare sensors requiring additional electronics. Range is presented as a fraction of the Lower Explosive Limit (LEL) which is typically defined as 4%_vol/vol_. Resolution details the minimum change in concentration reported by the sensor, although some fixed units provide only a binary alarm output should the range maximum be exceeded. Response time is defined as the interval required for a sensor to change its output from a stable background reading to the maximum change exhibited under hydrogen exposure. The beginning and end of this interval are defined as fractions of the final reading, with $t_{10-90}$—as typically reported in literature—representing the time taken for sensor output to vary from 10% of the final reading to 90% of that reading. Vendors do not apply these measurements universally and may instead report $t_{20-80}$ or $t_{50}$, which are generally less than $t_{10-90}$ and appear favourable. Few report the corresponding recovery time as returning the a background reading, listed as $t_{90-10}$. Certain sensor types require ambient oxygen to operate, and this is typical of electrochemical devices of metal oxides where the underlying physio-chemical interaction makes use of oxidation-reduction reactions. Where this is the case, the minimum allowable oxygen concentration is shown.

The most common technologies for use as hydrogen gas sensors are based upon electrochemical, catalytic or pellistor, thermal conductivity, and metal-oxide-sensing mechanisms. Other sensing technologies include pure metal, optical, and electromechanical sensors, which—while showing great potential for various applications—are not widely available commercially [44].

#### 4.1.1. Electrochemical Sensors

Electrochemical sensors are widely available for the detection of a range of chemical analytes including hydrogen gas. Typical electrochemical gas sensors consist of two or three electrodes and a liquid or solid phase electrolyte designed to operate in an amperometric (current) or potentiometric (voltage) mode [45]. Interaction between hydrogen gas molecules and the electrodes enables the charge transfer and generation of a small current or voltage (depending upon the operating mode) within the cell that can then be measured. A hydrophobic gas permeable membrane is typically included to both limit the diffusion rate of hydrogen into the cell, thus making current generation proportional to hydrogen concentration, and to mitigate the influence of humidity over sensor response. Liquid electrolytes are able to operate at room temperature whilst solid electrolytes enable detection in high-temperature environments up to approximately 1000${\;}^{\circ }C$. Electrochemical sensors are able to operate for months to years before requiring replacement, which is typically required due to degradation of the electrolyte over time. Electrochemical sensors require minimal power during both storage and operation, making them attractive for use in both fixed and portable applications. The shortcoming of this technology is moderate selectivity and a susceptibility to poisoning of the electrode or electrolyte, leading to permanent degradation in performance after exposure to select contaminants. Additionally, the underlying chemical interaction that enables charge transfer requires oxygen, making use in anaerobic environments infeasible.

#### 4.1.2. Catalytic/Pellistor Sensors

Catalytic or pellistor type gas sensors perform sensing by detecting the energy released during combustion of the targeted analyte. Typical construction centres around a resistive heating element coated in a noble-metal catalyst (usually platinum) which is heated to around the auto-ignition temperature of the target analyte; for hydrogen, this is approximately 550${\;}^{\circ }C$. Presence of the catalyst on the surface of the heating element enables localised combustion of the gas at the surface of the element, releasing heat energy that in turn affects the conductivity of the heating element. Change in conductivity of the element is therefore proportional to the rate of combustion, which in turn is proportional to gas concentration. To accommodate changes in operating conditions, a second element is typically integrated without a catalytic coating to serve as the reference for a bridge circuit, thus helping to isolate the local heating enabled by the presence of the catalyst. Catalytic gas sensors are simple in construction and—aside from the noble-metal catalyst—require inexpensive materials with minimal processing, making the sensors inexpensive, scalable, and robust. However, such sensors show poor selectivity as the sensing mechanism is excited by any combustible gas. Given the coexistence of the hydrogen economy alongside existing fossil-fuel consumption, this cross-sensitivity could lead to false detection in automotive and portable applications. Additionally, as the sensors require a high temperature to operate, there are substantial power requirements during operation.

#### 4.1.3. Thermal Conductivity

Thermal conductivity sensors also make use of thermal properties to infer gas concentration, albeit at a much lower temperature than that required for catalytic sensors. A resistive element of low thermal mass is supplied with a variable voltage, the current is measured, and from these parameters, resistance is characterised. This process results in the heating of the element which dissipates this heat to the surrounding media. As voltage is increased heat, is dissipated at an accelerated rate, and the observed resistance deviates from the ideal predicted by Ohm’s law (V=IR) by a factor proportional to thermal conductivity of the media. The thermal conductivity of hydrogen (0.187 W/(m K)) is much higher than that of air (0.026 W/(m K)), and therefore thermal energy is dissipated into a hydrogen-rich atmospheres faster than into air [39]. Such measurements are subject to changes in ambient conditions, and these can be compensated for through inclusion of a secondary reference sensor in a sealed container containing a reference gas, similar to the reference setup used with catalytic sensors. As there is no chemical interaction with the analyte, thermal conductivity sensors are quite durable, are able to operate with minimal drift for several years before replacement is necessary, and are resistant to poisoning by contaminant compounds. They are also able to work across a broad concentration range (up to 100%) without oxygen and at much lower power levels than those required by catalytic sensors. The shortcomings are poor sensitivity to low concentrations of hydrogen and the inability to differentiate hydrogen from other gases with thermal conductivity higher than that of air, including pure oxygen (0.027 W/(m K)), methane (0.034 W/(m K)), neon (0.494 W/(m K)), and helium (0.156 W/(m K)) [39].

#### 4.1.4. Metal-Oxide Sensors

Metal-oxide sensors (typically abbreviated to MOS or MOX) have long been discussed in the literature and are gradually becoming available on the general market. These devices utilise solid metal-oxide materials to perform sensing through various physio-chemical interactions, typically involving oxidation-reduction (redox) reactions utilising ambient oxygen [46]. Interaction with the analyte causes changes to the electrical properties of the semi-conducting metal-oxide, which is then measured quite trivially. There has been a recent focus on the application of nano-materials to such sensing devices, as nano-scale structures provide a substantial increase in the active sensing surface area and hence increase sensitivity. Sensitivity can be further enhanced through external optical stimulation of the material [47]. Selectivity can be achieved by incorporating multiple materials into hybrid structures.

#### 4.1.5. Other Technologies

Whilst several other technologies exist for hydrogen gas sensing, many of these currently have limited to no commercial presence. These include optical, pure-metal, and Micro-Electromechanical Systems (MEMS)-based sensing devices. Such technologies show good potential for esoteric applications such as in optical devices, which mitigate the risks associated with the use of electrical devices in and around explosive atmospheres. Others offer significant improvements to sensitivity or selectivity, as has been achieved with MEMS-based hydrogen sensors [44]. However, until such devices achieve widespread availability, which is problematic due to complex manufacturing requirements, materials cost, and lack of in-situ validation and testing, these advantages are of academic interest only.

### 4.2. Electric Sensors

Electric sensors are necessary to detect and measure the electrical characteristics (voltage and/or current) in power systems. These sensors translate potentially hazardous voltage or currents—such as those found in mains-grid-powered devices—into safe, low-level measurement outputs compatible with instrumentation, control equipment, and IoT devices. Depending upon application, the measurement output may be generated in one or more standard formats, including ±10 V analog voltage signalling, 4 to 20 mA analog current signalling, or a range of industrial digital communication buses (i.e., Modbus, Profibus, etc.).

#### 4.2.1. Voltage Measurements

Voltage sensing must reduce a high-voltage signal to a low-voltage one compatible with the connected equipment. This can be accomplished using resistors or capacitors to create a voltage divider. This approach is applicable to low-, medium-, and high- voltage systems, with maximum voltage dictated only by the division ratio and breakdown voltage of the components used. Additional electronics are typically employed to isolate the measurement output from the input to avoid cases where fault currents or voltages might be passed to sensitive measurement equipment.

#### 4.2.2. Current Measurements

Current sensing can be achieved using a number of different mechanisms. The simplest current sensor technology is the shunt resistor, in which the current flowing through the shunt results in a voltage drop as expressed via Ohm’s law ($V=I*R_{shunt}$). This voltage can them be measured directly or amplified and then measured as a voltage using one of the methods detailed above. However, this configuration results in power dissipation in the shunt and an associated temperature increase proportional to the current flow, making this approach undesirable in high-power systems. Additionally, the shunt induces a voltage offset (burden voltage) across the current sensor. The shunt is connected in series with a supply or load and can be placed at the high or low-voltage side of these connections, and each configuration has disadvantages. Low-side current sensing results in a measurement signal close to ground or 0 V; however, since 0 V is usually the reference voltage in any system, offsets can appear between equipment, potentially causing ground loops, introducing noise, and potentially tripping or impeding safety equipment. High-side current sensing avoids issues with the reference voltage in the system, but the measurement signal will be with respect to the voltage of the bus measured, and additional isolation may be required to bring this down to a safe reference level for other equipment.

Current can be measured non-intrusively via measurement of the magnetic field generated by current flow. This field can be measured by a current transformer, a magnetic element specially designed to interact with fields radiated by current flow and translate them to a voltage output. These transforms only operate with AC systems, as a varying waveform is necessary to generate a magnetic field. Increasingly, hall effect sensors are being used for current measurement as they are non-intrusive and isolated and are capable of measuring currents from mAto MA Certain magnetic material properties can additionally be exploited to generate current measurements using active Hall effect sensors in DC systems although such sensors are typically more expensive than are other types.

#### 4.2.3. Additional Measurements

A combination of voltage and current measurements can be used to calculate a number of other parameters important in electrical systems, such as real and apparent power and power factor and lead/lag angle. These variables are typically calculated electronically in modern systems. AC systems are additionally classified by a number of temporal and spectral measurements that require more complex equipment to capture and quantify. These include AC frequency (typically 50 Hz or 60 Hz depending upon country) and the magnitude and frequency of harmonics.

### 4.3. Internet of Things

**Figure 2:**
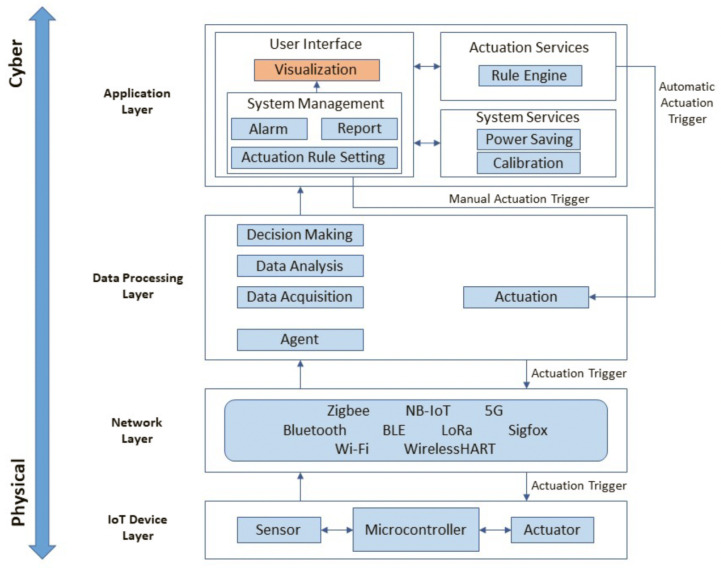
An overview of a typical IoT architecture in Hydrogen 4.0.

The emergence of IoT devices and technologies and the capabilities they enable have opened new horizons for monitoring and optimising various industrial processes, including in hydrogen plants. IoT is defined as technology that interconnects billions of sensors and other devices (i.e., things) via the Internet, enabling novel services and products [48]. IoT enables automation in data collection, data analysis, decision-making, and actuation processes and acts as a complementary technological element for Machine Learning (ML) and AI models by enabling the collection of operating data. These data are a critical resource in the development and testing of advanced models in these fields, improving decision accuracy, process efficiency, and effectiveness. The IoT can additionally facilitate reactive responses to risks in plants by enabling real-time monitoring capabilities and aid in proactive risk management by predicting potential issues. A typical IoT architecture is illustrated in Figure 2. The architecture is logically divided into four layers, including (i) devices, (ii) networks, (iii) data processing, and (iv) application, with actuation processes creating feedback loops between the layers, and thus the impact of actions by theIoT environment is constantly self-monitored by the environment. In the following sections, each layer of the IoT architecture will be discussed in detail.

#### 4.3.1. Device Layer

**Figure 3:**
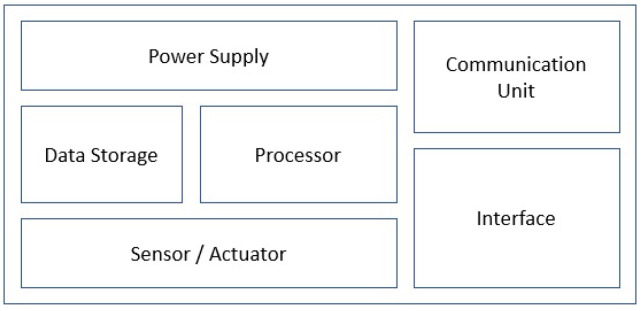
Block diagram of a typical IoT device.

IoT devices typically are miniature computing devices centred around a microcontroller or microprocessor that accommodate the minimal system requirements to interface to sensors, actuate external equipment, and communicate with data collection and control infrastructure via an internet connection. Figure 3 provides a block diagram of such a device.

IoT devices have several constraints including power source, processing power, memory, connectivity, enclosure design, security, compatibility, and cost. Each of these constraints is discussed in detail below:

- Power source: IoT devices are spatially distributed and used to collect data from a variety of equipment and locations in plants, potentially across different geographical locations. This can make it impractical to power all the IoT devices in a network from mains power. Therefore, alternative energy sources play a crucial role in IoT device design and deployment. Battery-powered devices are widely available, although they come with limitations on lifetime and require periodic maintenance. Battery-powered IoT devices often implement a range of operating modes to extend battery life by dropping to low-power sleep mode, which disables components not in use. Waking from sleep can be triggered periodically (by an internal timer) or by an external input, with waking occurring only as required to collect and transmit measurements. The ratio of active to sleep time is called duty cycle, and by spending the majority of the time in sleep mode, a battery can extend its life. Other parameters, such as choice of network technology and topology, can influence the minimum average power usage. As an example, use of short-range radio links and a mesh topology may reduce power usage compared to a long-range radio, but nodes may need to be active for longer periods to ensure messages are passed through the mesh without being dropped.
- Processing power: In settings where available electrical power may be limited (as above), it is preferable to optimise the computing capabilities of IoT devices. Typically, such devices facilitate only the collection and distribution of data, and as such, have minimal requirements in terms of computational power. Low-power micro-controllers or microprocessors are attractive for use in IoT devices owing to their minimal power draw, integrated peripherals, and small physical footprint. Where more complex computation is needed, this is typically handled at another layer (discussed in Figure 2) where computationally intensive data processing and decision-making processes are conducted. However, this has the drawback that latency can be introduced between sensing and actuation, and system complexity may increase due to increased reliance on higher layers [49,50].
- Memory: Similar to processing power, the compactness and energy efficiency of IoT devices also impacts their memory size. Most IoT devices have limited memory which limits the types and quantity of data that can be processed. Video and image data in particular can have substantial memory requirements, limiting the update rate or resolution of visual information. Acquisition of signals with high-bandwidth (and hence high sample rate) may be similarly limited by local device memory. This can be overcome by streaming data to a high layer, although this makes it more susceptible to data loss if the IoT network is unstable.
- Connectivity: IoT Devices utilise the Internet as a backbone for command and control, as well as data transmission. Internet access can be limited in remote locations, and therefore, implementing IoT devices in such locations can be challenging. There are a number of existing and emerging standards that enable connection to internet services using long- and medium-range wireless protocols. It is worth mentioning that hydrogen plants are usually far from residential and commercial buildings, which can pose a challenge in regards to internet access provision.
- Enclosure: IoT devices can be deployed in environments that are hostile to electrical systems, such as outdoor environments, or may include exposure to toxic, explosive, or corrosive atmospheres. In addition, environmental exposure can cause sensor drift due to water vapour or dust. This can result in noisy data, which can in turn reduce the efficiency of data analysis and the decision-making process. Enclosure selection enables IoT devices to be deployed in such environments without damage.
- Security: Limited processing power and memory makes it challenging to implement security measures on IoT devices and results in security risks on this layer of IoT environment. These risks include hacking, malware attack, data breaches, and unauthorised access. Such security threats can be extremely critical when IoT is deployed in sensitive sectors such as hydrogen plants. As a result, security and access control measures are among the main concerns in systems including IoT devices and must to be addressed with high priority by implementing IoT-based effective security techniques and algorithms [51].
- Compatibility: Since IoT devices are meant to collect data from various aspects of the environment, there i as need to use different components with heterogeneous characteristics. This results in compatibility issues of IoT devices from different perspectives including hardware, software, data formats, and standards. IoT devices can have different communication protocols and also collect different types of data, such as optical or numerical data [52]. Such heterogeneous characteristics of IoT devices can make the data aggregation and integration a challenging task, which can result in the reduced efficiency of the decision-making and actuation process. Addressing the compatibility issue is the responsibility of manufactures and developers to create common standards, protocols, and interfaces for IoT devices.
- Cost: The cost of procuring, deploying, and maintaining a network of IoT devices is a major constraint on plant design. Since some data types are spatially sensitive and need to be collected across different geographical locations, the number of required of IoT devices can increase, which can significantly increase the cost factor. Networking the IoT devices into a centralised cloud-based platform can also be cost intensive at both the development and maintenance stages. In addition, addressing different issues such as latency and security can further increase the cost of IoT device procurement where more powerful hardware and software components must be acquired. Therefore there is a tradeoff between cost and other constraints that must be taken into consideration when designing an IoT architecture for the hydrogen plants.

#### 4.3.2. Network Layer

Connectivity between IoT devices and infrastructure on highers layers is conducted on the network layer. There are multiple communication protocols widely utilised in IoT architectures including Long-Term Evolution (LTE), Narrowband Internet of Things (NB-IoT), LoRa, Bluetooth, Bluetooth Low-Energy (BLE), ZigBee, and others. Certain legacy protocols (e.g., Modbus/RTU) have compatibility with modern networks (e.g., Modbus/TCP). Table 2 provides an overview of several such protocols and summarises some of the characteristics important for the selection of a protocol for an IoT application.

The common backbone to all IoT protocols is the bridging communication to the global internet at one or more layers to facilitate communication between IoT devices and devices at higher layers of the architecture. Each protocol has advantages and disadvantages depending upon the specific application. Selection of a particular communication protocol depends on the IoT devices in use, deployment topology, data type and collection frequency, and required communication distance. A single IoT architecture may use multiple communication protocols in different segments of the network if required to fulfil specific requirements. For example, in an application where IoT devices must communicate with one another locally, a low-range protocol such as Bluetooth or WiFi might be employed in addition to a secondary LoRa or NB-IoT interface for transmission of data to edge devices.

Plant data often have commercial value or contain sensitive information, and as such, the security aspects of communication protocols play a major role in the selection process. WirelessHighway Addressable Remote Transducer (HART), LoRa, and NB-IoT are among the secure protocols which are suitable for hydrogen plants. WirelessHART is a suitable choice for communication of IoT devices in a short distance. NB-IoT and LoRa are more suitable for for longer range communications up to several kilometres, which can be necessary for monitoring tanks and pipelines in the hydrogen plants.

#### 4.3.3. Data-Processing Layer

Given the limited processing ability of IoT devices, data processing in IoT deployments is typically carried out at the edge or using cloud resources. Although it is not necessary to integrate edge computing into all IoT applications, there are several benefits to integrating some processing into local resources, particularly in a hydrogen plant. Edge computing reduces the latency between devices and decision-making and enables systems to more rapidly respond to sensor data, which can be critical in leak detection or equipment failure detection. In addition, in applications where data sensitivity and security is a concern, including edge computing can confine highly sensitive data to the local facility, decreasing the potential attack surface and value of—generally more exposed—cloud resources. Furthermore, edge computing can act as a backup when cloud resources are unavailable due to service interruptions or unreliable connections.

The complexity of an end-to -end energy life cycle including energy production, storage. and distribution makes data analytics in this industry a challenging task. Interconnection of multiple subsystems with heterogeneous data from heterogeneous sensors requires advanced data analytic applications to efficiently translate the acquired data into meaningful information. Some of the existing data analytics used to address such challenges in digitised energy sectors are Databricks, Metabase, Snowflake, and Azure Synapse Analytic. Databricks is an open analytic platform for building, deploying, sharing, and maintaining data. It enables data processing management, data discovery, data ingestion, data annotation, and machine learning modelling. One of the advantages of Databricks is its capability to be integrated with customer cloud services such as AWS and therefore does not require data migration for implementation [53]. Metabase is another data analytics tool with a focus on simplifying the data analytic process and enabling the customers to perform their data analysis on their own. Metabase provides an outstanding and easy-to-use dashboard which facilitates the data visualisation and development of semantic models to improve the decision-making process [54]. Snowflake is a cloud-native analytics tool which enables data analytics for businesses with different scales and data integration from multiple cloud sources or between cloud and am on-premise environment. Azure Synapse is another analytics tools which enables large-scale data analysis of data warehouse and bid data repositories [55]. It focuses on end-to-end analytics solution, which makes it a potential analytics tool suitable for addressing the complexities of interconnected subsystems in the energy industry [56].

The Hydrogen 4.0 paradigm takes full advantage of the powerful data analytics capabilities enabled by cloud resources by applying contextualisation, approximation, machine learning, and other AI techniques to data acquired via the IoT device layer [57,58]. Such data analysis can significantly improve power plant’s management process through automaton and optimisation of tasks such as predictive maintenance, security monitoring, collaboration between stakeholders, and automatic operation.

#### 4.3.4. Application Layer

The application later is where results of data processing are translated into visualisations and key decisions are made. The analytic capabilities of the application layer enable plant owners and operators to improve their decision-making efficiency through use of different analytical tools such as machine learning or statistical models. This is coupled with rule-based automated decision-making that performs actions autonomously in response to plant data and information gathered from external resources (e.g., weather service data). In addition, users can perform manual actuation—if necessary—by exerting control manual control over IoT devices. Role-Based Access Control (RBAC) can be employed to provide authentication and authorisation of users, reducing access to sensitive data and actuation tasks [51,59]. Management of cloud infrastructure and deploying software and/or firmware updates to IoT and edge devices are also facilitated at this layer. Automated reporting and alarms can be configured depending upon the safety, regulatory, and business needs of a Hydrogen 4.0 plant.

### 4.4. Cloud Providers

There are currently numerous IoT platforms in the market which are well-suited for use with IoT devices in hydrogen plants. Some of these platforms include OpenRemote, ThingSpeak, Amazon Web Services (AWS) IoT Core, Microsoft Azure, and Google Cloud [60].

OpenRemote is one of the leading IoT platforms in the energy management sector. It is an open source platform which is free to use. One the main advantages of the OpenRemote IoT platform is it capability to support a wide range of communication protocols (e.g., Hypertext Transfer Protocol (HTTP), Transmission Control Protocol (TCP), User Datagram Protocol (UDP), Websocket, Message Queue Telemetry Transport (MQTT), and Modbus) which makes it an outstanding choice for use in hydrogen plants where there are heterogeneous devices from different vendors with different communication requirements. In addition, it is integrated with a rule engine to automate the data flow and actuation processes. Another advantage of the OpenRemote IoT platform is the fact that it is simple to use and does not require coding knowledge to implement.

AWS IoT Core is another IoT platform which supports multiple communication protocols including WebSocket, HTTP 1.1 and MQTT. AWS IoT Core addresses the security issue by performing mutual authentication and end-to-end encryption. The authentication methods of AWS IoT core include SigV4 authentication, X.509 certificate-based authentication, and customer-created token-based authentication. This is a very beneficial feature for plants where cyber-attacks can cause devastating damages. In addition, AWS IoT Core integrates machine learning capability to enhance data analysis, decision-making, and the actuation process [61].

Microsoft Azure is another IoT platform which is widely used. One of the main advantages of Microsoft Azure is high availability. High availability is of high priority for sensitive industries such as hydrogen plants which require constant monitoring and management. In addition, Microsoft Azure also uses several encryption technologies to improve its security.

Google cloud, along with AWS IoT Core and Microsoft Azure, is among the top three IoT platform vendors [62]. Google cloud provides one of the most secure IoT platforms by using different security services including Transport Layer Security (TLS) for device-cloud encryption, Java-Script Object Notation (JSON) web tokens, X.509 certificate-based authentication, Elliptic Curve Cryptography (ECC) and Rivest-Shamir-Adleman (RSA) encryption [62]. Google cloud also has an uptime of ≈ 99.5%.

Another major IoT platform which has attracted much attention in recent years is ThingSpeak. ThingSpeak was developed by MathWorks to integrate their popular data analysis tool, MATLAB, with IoT device data. MATLAB provides a number of data visualisation, analysis, and decision-making tools and is popular amongst engineering professionals. In addition, ThingSpeak provides a rule-based IoT platform which can automate actuation based upon acquired information.

### 4.5. Digital Twins

The hydrogen supply chain is a complex ecosystem with numerous variables that must be considered to achieve peak efficiency and effectiveness. Optimising the supply chain can contribute to hydrogen price reductions by achieving equilibrium between supply and demand. However, the complexity and safety implications of experimenting on real-world plant equipment makes the pilot testing of new processes prohibitive. DTs provides an alternative platform for such experiments, providing a highly accurate facsimile of real-world equipment in a virtual sandbox environment. The DT concept consists of three main parts: (i) a physical system in real space (physical twin), (ii) a virtual system in cyberspace (DT), and (iii) an interconnect between the cyber and real space to provide up-to-date plant data via IoT systems [63]. This enables the comparative study between the cyber and physical representations of the plant, providing a feedback loop to better refine the digital model and an early indication of faults if unexpected deviations develop between the two plants.

Xe et al. [64] applied DT modelling on a power plant for performance optimisation. They reported that with a DT modelling approach, they were able to reduce the boiler exit-flue-gas temperature by up to 25${\;}^{\circ }C$ from normal levels, leading to an improvement in plant heat rate. These findings indicate that at large scale, DT modelling can result in significant capital saving. The vast amount of data generated by DTs can be used for training machine learning models to identify supply chain bottlenecks and formulate new processes and improve supply chain efficiency. This removes the risk of pilot testing new processes on real-world plant equipment where potential optimised processes are being implemented and tested in a well-developed DT environment.

A well-developed DT can also be used to look forward in time and predict future maintenance requirements and anticipate component failure [6]. This can extend equipment longevity and minimise running costs and downtime by optimising maintenance window duration whilst maximising efficacy. DTs can help to identify the early signs of equipment degradation and abnormal behaviour and propose required maintenance before such anomalies would interrupt the plant operations. Real-time monitoring enables ongoing collection of equipment performance metrics and environmental parameters, allowing for the detection of operational anomalies whilst providing a cohesive overview of plant and equipment run time, load, and operation conditions. In addition, through using historical data and machine learning models, DTs can improve predictive analytic and forecast future equipment behaviour, subsequently estimating optimal timing for regular maintenance cycles.

Last but not least, the DTs enable the application of what-if scenarios to evaluate the impact of different scenarios on the hydrogen plant process and to formulate efficient predictive maintenance procedures, resource allocation, and optimisation strategies to address the potential risks for each scenario. As an example, DTs enable simulation of Integrated Gasification Combined Cycle (IGCC), which is an advanced approach for power generation modelling involving multiple feed-stocks [65]. IGCC can produce energy with higher efficiency and lower emission compared to conventional coal-fired power plants. IGCC can be also used in hydrogen plants and can significantly improve the efficiency of the power generation process through DT modelling, something challenging to implement without a DT to experiment upon.

Having discussed the potential advantages of DTs in improving energy production sector from different aspects including supply chain process optimisation, predictive maintenance, cyber security improvement, and risk management, in the following section, we turn to some of the leading DTs that can provide these capabilities for energy sector and discuss them in detail.

#### Digital Twin Platforms

The DT market is expected to grow from USD 10 billion in 2023 to USD 110 billion in 2028 [66]. Such a substantial leap in market share of DT indicates that the adoption rate of DT by companies around the world is exponentially on the increase. This market has attracted some major players including General Electric, Microsoft, Siemens, Amazon, and BOSCH [67]. These DT service providers enable the companies around the world to improve their business from different perspectives, including predictive maintenance, mean time between failures (MTBF), mean time to repair (MTTR), product design and development, business optimisation, inventory management, and quality improvement [66]. The energy industry is among the main sectors with the highest adoption of DT applications. The energy sector operates based on the interconnection of complex systems. Managing such a complex network of interconnected systems is a challenging task, where a huge number of data points and their correlation need to be analysed to efficiently optimise the existing processes. DTs provide a substantial solution for the analysis of such complex systems in which heterogeneous data across different energy life cycle systems can be integrated and analysed [8]. This approach helps make data-driven decisions, enhance operational efficiency, and ensure a reliable and sustainable energy supply chain for the future. In the following section, some of the main DT platforms that can be used to address the complexity of the energy sector are discussed.

General Electric, which is one of the leading DT providers, offers a wide range of services for DT life cycle including DT implementation and management, data collection, data analysis, and consultation services to help the businesses benefit from DT capabilities without facing the challenges in using such systems. They provide DT services for a wide range of industries, including renewable energy, aviation, manufacturing, and healthcare [68]. General Electric claims that their platform improves the reliability 93–99.49%, reduces the reactive maintenance by 40% in less than 1 year, and reduces the time to achieve outcome by 75%. They have 330 DT blue prints to be used instantly across different industries, which has resulted in savings of USD 1.6 billion for the clients to date [68].

Microsoft is one of the leading companies in providing end-to-end DT services from data collection to data analytics in the energy sector, aiming to materialise the net zero emissions objective. Microsoft integrates Azure Digital Twin service with its Azure IoT platform and Azure Synapse analysts to enable energy production companies to seamlessly collect data from a wide range of heterogeneous senors and turn these data into useful insights to improve energy production processes. In 2022, Microsoft with cooperation with Capgemini integrated a cloud-native serverless DT platform in Azure called ReflectIoD. ReflectIoD is an open source DT platform which focuses on Smart data management, intelligent monitoring, and augmented maintenance [62]. In addition to analysing the live data, Azure DT also focuses on integrating historical data into its data repository to identify the long-term trends and help to optimise the energy production, storage, and distribution processes based on both historical and real-time data. Such an approach helps to provide a macro level of understanding regarding the energy supply chain and enables the formulation of new processes to improve the efficiency and effectiveness of energy supply chain management [69].

Siemens is another company which offer a wide range of DT services for the energy production sector [4]. The Siemens Digital Enterprise Suite provides DT services in term of DT of product, production, and performance. The DT for products focuses on the product attributes and helps the engineers to simulate and validate the product properties on the respective requirements. LeBlanc and Ferreira [70] used experimental modal analysis (EMA) to enhance the accuracy of a digital twin model of an H-VAWT turbine. This technology helped in validating and refining the model by aligning it closely with the physical dynamics of the turbine through detailed testing and data correlation. The precise matching of the digital twin with real-world performance data enabled effective optimisation of the turbine’s design and functionality. The DT of production focuses on the devices which are used in the production process in a virtual environment which can help to improve the production optimisation. The DT of performance focuses on operational data to analyse the energy and cost spent to produce the final product and offers new processes to improve the production efficiency and effectiveness. Such wide range of DT features can provide real-time data analysis regarding the energy production, the devices used in the energy production process, and the performance of energy production processes, which can lead to a highly efficient energy production supply chain. Siemens also employs the defence-in-depth strategy to address the security issue of the DTs. This strategy uses a standard compliant security mechanism and constant security monitoring to minimise the cyber security risks to the DT [71].

Amazon IoT Twinmaker is another leading DT platform which enables the data collection from a diversity if data sources, including IoT data, video data, application data and event data [62]. Some of the main features of Amazon IoT Twinmaker that enable for the addressing of the complexity of end-to-end energy supply chain management are data connectors, model builder, scene composer, and applications. Twinmaker provides built-in data connectors including AWS IoT SiteWise to collect and organise time-series sensor data and Amazon Kinesis Video Streams for capturing, processing, and storing video data. The model builder feature facilitates creating entities and identifying their relationship to develop a virtual representation of the energy physical system. The scene composer feature enables the development of a 3D DT, and the applications feature enables a low-code experience web application development that provides the engineers and managers with real-time access to the power plant DT environment [72].

The Bosch Digital Twin platform provides multiple delivery methods for its DT services including, on-premise, software as a service (SAAS), platform as a service (PAAS), and infrastructure as a service (IAAS). They provide an AI-powered DT platform which enables the data collection and analysis of heterogeneous data sources spread across a large span of geographical locations where energy sector subsystems are connected to streamline the production, storage, and distributions processes. Bosch Digital Twin enables for the prediction of problems, using automation and recommending solutions to reduce maintenance costs and downtime while improving the business margins. Thee main objectives of providing such services are to automate and eliminate repetitive task—with data being used for better decision-making making—reduce uncertainties and risk, and enable pre-emptive problem-solving [73].

General Electric, Microsoft, Siemens, Amazon IoT, and Bosch offer sophisticated digital twin technologies tailored to diverse industrial and commercial applications, each with distinct strengths. Siemens excels in integrating complex systems for sectors like energy, focusing on performance and operational reliability, a capability well illustrated through their use in wind turbine optimisations. General Electric’s Predix platform also targets industrial applications, providing robust data analytics to improve operational efficiency. Microsoft Azure facilitates broader application scenarios, using its cloud infrastructure for scalable digital twin implementations across various industries. Amazon’s AWS IoT TwinMaker simplifies the development of digital twins in the cloud, making it accessible for IoT-focused projects and smart city applications. Bosch, with a strong focus on automotive and manufacturing industries, integrates real-time sensor data with digital replicas to enhance predictive maintenance and operational decisions. Collectively, these platforms showcase a range of capabilities from detailed engineering simulations to cloud-based solutions, supporting a wide spectrum of digital transformation strategies across industries [62,74].

In addition to the aforementioned key players in the DT market, there are other main companies which concentrate on providing DT services. Some of these companies include Oracle, Cisco, IBM, ANSYS, Dassault Systèmes, PTC, and SAP. While these providers offer powerful digital twin platforms that cater to a variety of industrial and commercial needs, these systems are not necessarily tailor-made for energy plants and may require significant modifications to meet the specific demands of the energy sector. Energy plants feature unique complexities, including the need for highly specialised simulations of energy flows, regulatory compliance, and integration with legacy systems. Therefore, although these digital twin platforms offer a strong foundation with their advanced analytics and modelling capabilities, customising them to address the precise operational challenges and safety requirements of energy plants is essential. This customisation might involve developing additional modules to handle the specific types of data generated by energy systems or integration with specialised tools that are already in use within the energy sector. Such modifications ensure that the digital twin effectively contributes to enhancing operational efficiency, predictive maintenance, and system reliability specific to the energy industry’s needs.

## 5. Hydrogen 4.0 Application Scenarios

Hydrogen 4.0 represents an innovative integration of Industry 4.0 technologies, including the IoT and DTs, specifically tailored to enhance the hydrogen production industry. This concept builds upon the existing framework of cyber–physical systems to create a more interconnected and intelligent infrastructure within hydrogen plants. By embedding smart sensors and implementing advanced data analytics, Hydrogen 4.0 aims to improve operational efficiency, safety, and compliance across all aspects of hydrogen production and distribution.

This section will present a number of applications of the Hydrogen 4.0 paradigm, and present the potential benefits to each enabled via implementation of technologies including sensors, IoT, DTs, and edge and cloud computing resources.

### 5.1. Energy Production Efficiency

IoT has the potential to improve energy production efficiency in hydrogen plants via several avenues. Firstly, by enabling predictive maintenance, the potential downtime of hydrogen plants can be minimised, and thus energy production will not be interrupted. This can be coupled with data from external sources, such as weather forecasts, to schedule maintenance in windows where production would be minimal due to unfavourable operating conditions. Additionally, IoT connectivity enables real-time monitoring that can aid in online process optimisation and tuning, as key parameters such as temperature, pressure, and flow can be recorded and visualised with minimal delay.

DTs allow virtual replication of plant equipment and resources and can act as an experimental platforms for process tuning. This can help identify production bottlenecks and increase overall production. Moreover, since the energy used by hydrogen plants can originate from different sources—such as renewable energy, biomass, and fossil fuels—both IoT technologies and DTs can be used to optimise energy delivery and use. IoT, in particular, can be used to coordinate backup operations and automated handover during maintenance windows when energy supplies are interrupted (e.g., drops in local solar or wind power) or when operation would not be economic. Thus, both IoT and DTs have the potential to improve energy efficiency of hydrogen plants and ensure the availability and reliability of the energy delivered to and by such plants.

### 5.2. Safety & Compliance Monitoring

Hydrogen gas is an extremely volatile flammable and explosive compound that requires special measures to guarantee safe handling, and thus safety measures are among the main concerns in any hydrogen facility. The primary hazards associated with hydrogen are combustion, high-pressure, low or cryogenic temperature, hydrogen embrittlement of metal pipes and components, suffocation, and exposure [75]. Real-time data collection and monitoring from various entities within hydrogen plants via IoT devices can assist in ensuring the safe operation of the plant and safety of plant personnel. The location and state of entities including pipelines, tanks, valves, and plant personnel can assist in the event of an emergency or evacuation.

IoT devices provide both reactive and proactive safety and compliance monitoring capabilities. IoT can provide real-time data with high precision to help identify potential hazards within the plant. In case of hydrogen leak detection, IoT can trigger automated shutdown to prevent the damages to plant infrastructure and personnel. From a proactive perspective, IoT can enable predictive maintenance through constant monitoring of hydrogen plant infrastructure. The analytic capabilities enabled by IoT systems have the potential to predict future system failure and alert the plant managers before risks turn into potential hazards.

### 5.3. Green Transport

Greenhouse gas emissions and global warming has shifted the attention of the developed world toward green transport. Introduction of Fuel-Cell Electric Vehicless (FCEVs) using hydrogen-based energy carriers has opened new horizon for decarbonising the transport sector [76,77]. A key challenge faced by the infrastructure to support FCEVs is keeping an equilibrium between supply and demand to ensure constant hydrogen fuel availability whilst minimising stored hydrogen. Digitalisation of hydrogen plants and hydrogen refuelling stations using IoT enables a bidirectional data exchange between these two sectors to achieve such equilibrium. The IoT enables real-time demand monitoring by hydrogen plants which can help to optimise the production quantity. In addition, refuelling stations can be informed in real-time regarding fuel availability and be informed of delays in the supply chain. The IoT can streamline the ordering process for refuelling stations and improve supply and demand efficiency. In addition, FCEVs can also access real-time data regarding refuelling stations in their vicinity and the fuel availability. Such integration enables an end-to-end Hydrogen 4.0 supply chain, facilitating a transition towards reliable and efficient green transport.

### 5.4. Green Hydrogen Supply Chain

Hydrogen supply chains consist of multiple layers including selection of energy source and hydrogen production, transportation, storage, and utilisation technologies. Each layer within this stack can utilise different types of technologies. Energy may be sourced from renewable sources, fossil fuels, biomass, nuclear, or other sources. The hydrogen production may be achieved via SMR, electrolysis, or gasification, to name several popular technologies. Transportation could be accomplished through pipeline or tube trailers. Finally, the hydrogen may be exported as a product, used locally for energy storage, used as a transport fuel, or employed for heating and cooling systems [76]. This demonstrates that the hydrogen supply chain is diverse and that plant design and operation can vary significantly from one application to another. This adds complexity to hydrogen supply chain management. However, integration of the IoT across different layers of supply chain can help to monitor and optimise such processes [78,79]. Hydrogen 4.0 can benefit from the communication between different sectors and help predict supply and demand patterns, optimising distribution and utilisation.

As previously discussed, there are a number of safety and compliance concerns around deploying hydrogen as a public resource. The complexity and hazards of infrastructure required to produce, deliver, and utilise hydrogen is greater than that of the technologies hydrogen is poised to replace, such as petrochemical fuels. This is further complicated by the diversity of hydrogen plants, making development of universal solutions via conventional system analysis infeasible and requiring unique solutions for each plant, increasing capital cost. An avenue to optimise the distribution of hydrogen plants across large geographical areas is enabled by the IoT, as it provides a real-time overview of hydrogen demand. Thus, plants and refuelling stations can be strategically deployed in areas of greatest demand and delivery routes can be optimised to enable just-in-time delivery. This minimises the energy consumption during transport and also reduces overall safety and financial risks.

### 5.5. Green Manufacturing

Green manufacturing utilises energy from renewable resources and feed-stocks to minimise the carbon footprint associated with the production of consumer and industrial products. Hydrogen 4.0 can be a key intermediary in this paradigm by providing an energy storage medium or providing a just-in-time mechanism to match energy supply and demand. This significantly improves the reliability of manufacturing capacity that is contingent upon the ongoing availability of cheap and accessible renewable energy whilst remaining sustainable. Afgan and Carvalho [80] assessed the sustainability hydrogen energy systems and noted that a focus on performance, environment, and market indicators was key to decision-making in such plants. Hydrogen 4.0 can provide a valuable infrastructure to collect data regarding such indicators and assist in the decision-making process. This has the potential to enhance the economics surrounding green manufacturing and extend its capabilities and product scope.

## 6. Conclusions

The exponential growth in market demand within the next three decades highlights the essential role hydrogen will play in the global energy landscape. Currently, hydrogen production mainly relies on established methods such as natural gas reforming and coal gasification, with only a minimal four percent contributed by green energy sources, such as electrolysis utilising solar energy or any alternative renewable energy source. This paper identifies key challenges in transitioning to green hydrogen, discussing issues related to production efficiency, equipment maintenance, compliance monitoring complexities, and safety concerns. The term Hydrogen 4.0 is introduced as an intersection of renewable power plants with digitalisation technologies such as IoT and DT, leveraging the digital capabilities derived from Industry 4.0. This paper outlines and discusses Hydrogen 4.0 in respect to an example of a renewable hydrogen plant and presents potential application scenarios.

The future direction of this work is to design and develop an implementation and evaluation framework for Hydrogen 4.0. The future for this paradigm appears bright. Whilst disruptions caused by maintenance or upgrade work to introduce novel technologies into established industries can be seen as too risky, the evolving hydrogen economy presents a unique opportunity to implement the tenants of Industry 4.0 from the outset, potentially leading to widespread adoption. This will hinge upon the development and distribution of industry standards at all layers, including schema for data collection and analysis, specifications for hardware and software interfaces to plant equipment for command and control, security and verification requirements, and algorithms for optimal plant operation, particularly across multiple geographically separate facilities. This is an area of ongoing development and research, and collaboration between academia and industry presents an optimistic future for uptake of the Hydrogen 4.0 paradigm.

## Figures and Tables

**Table 1:** Survey of commercially available hydrogen gas sensors targeted towards safety applications.

Name	Format	Type	Range		Res. *	Response Time ${\;}^{†}$				Temperature		Humidity			Service Life			O_2_ ${\;}^{§}$	Ex. ${\;}^{¶}$	Cost
			**%**_**LEL**_		**ppm**	**Seconds**				${\;}^{\circ }$ **C**		**%**_**RH**_			**Months**					
			**Min.**	**Max.**		$t_{50}$	$t_{20-80}$	$t_{10-90}$	$t_{90-10}$	**Min.**	**Max.**	**Min.**	**Max.**	**Cond.** ${\;}^{‡}$	**Min.**	**Typ.**	**Max.**			
Aeroqual Hydrogen (H2) Sensor Head	Sensor	MOx	0.01	12.5	1			30		0	40	10	95							High
Amphenol SGX Sensortech EC4-1000-H2	Sensor	EC	0	2.5	10			70		−20	50	15	90	No		6				Mid
Amphenol SGX Sensortech SGX-BLD1	Sensor		0	250			0.06			−40	85	0	95		18					Low
Amphenol SGX Sensortech TC1326-AS	Sensor	TC	0	0								0	95	No						Low
Amphenol SGX Sensortech VQ21TSB	Sensor	Catalytic	0	100				2												Low
CTI GG-H2-EC-10000	Fixed	EC	0	25		10		30		−30	50	5	100	Yes		24				High
CTI GG-H2-EC-10000-EXP	Fixed	EC	0	25		30		60		−20	50	5	100	No	24		60		✔	High
CTI GG-LEL2-H2	Fixed	Catalytic	0	100		20		45		−40	60	5	100	Yes			24		✔	High
Detcon DM-700-H2	Fixed	EC	0	100				60		−35	55	5	90	No			24		✔	Mid
ESP Safety Vector 100-0008-A1/100-0015-H2-1	Fixed		0	100		40		60		−50	75	0	100	No			60		✔	Very High
Airmet Gasbadge Pro	Portable		0	5	1														✔	Mid
Honeywell Sieger Sensepoint	Sensor	EC	0	25		10		45		−15	40	20	90	No		18			✔	High
Honeywell XCD Sensepoint	Fixed	EC	0	2.5				65		−20	55								✔	Very High
Honeywell XNX Universal	Fixed	EC	0	25		15		30		−20	55	15	90	No		24			✔	Very High
Honeywell Zareba Sensepoint	Fixed		0	25						−5	40	20	90		24		60		✔	High
Hydroknowz	Fixed	Catalytic	0	62.5	100			2	5	−40	80	0	99	No				>5%		Mid
Various MQ-8	Sensor	MOx	0.25	25																Low
Macurco EX-H2-2000P-O	Fixed	EC	0	5						−40	70	5	95	No			12			Very High
Macurco HD-11	Fixed	MOx	0	10	Binary ${\;}^{1}$					0	50				84		120			Mid
Macurco X-H2-100L-O	Fixed	Catalytic	0	100						−40	70	5	95	No			12			High
Oldham OLCT 10	Fixed	Catalytic	0	0		10				−10	45	0	95	No	36		48		✔	Mid
Orbisphere 29015A	Sensor	EC	0	0.08				50		−5	10									
Orbisphere 31290	Sensor	TC	0	0.05				17		0	50				6					
POSIFA Technologies PGS1000	Sensor	TC	0	100	1			2		−40	85									
PROSENSE P-3634	Fixed	Catalytic	0	5				20		−40	70	5	95						✔	Mid
Panterra Hydrogen Sensor	Fixed	TC	0	2500														None		High
SGT-P	Portable	EC	0	2.5	5					−40	50	5	95	No	24		60		✔	
PowerKnowz Hydrogen Sensor	Fixed	MOx	0	50	100			4	10	−40	80	10	95	No				>10%		Mid
Protisen Hydrogen Sensor	Fixed	Catalytic	0	100	200			2	5	−40	80	0	99	No				>5%		
Renesas SGAS701	Sensor	Chemi-resistor	0.025	2.5				15		−20	50	0	90	No						Mid
SBS-H2	Fixed	MOx	0	50	Binary ${\;}^{1}$					0	50	20	95	No	12		36	>0% ${\;}^{2}$		High
Simtronics Multitox DG-TT7-E	Fixed	EC	0	5				70		−20	50	0	99	No			24		✔	
Simtronics Multitox DG-TX7	Fixed	Catalytic	0	100				15		−40	60	0	99	No			60		✔	
TOC-30-H2	Fixed	EC	0	2.5				30		−5	50	5	95	No						
TOCSIN 903	Fixed		0	2.5				30		−10	55	20	90	No	60				✔	
mPower UNI MP100	Portable	EC	0.03	5	10			70		−40	50	5	95	No			12	>1%	✔	Mid

* Resolution: minimum step between measurable/displayed concentrations. ${\;}^{†}$ Recovery time. ${\;}^{‡}$ Operation in condensing humidity (≥100%_RH_) permissible. ${\;}^{§}$ Oxygen required for operation, with the minimum concentration being listed. ${\;}^{¶}$ Rated for operation in explosive atmospheres (ATEx or IECEx). ${\;}^{1}$ Alarm or relay output triggered at concentrations above maximum concentration listed. ${\;}^{2}$ Minimum concentration not specified.

**Table 2:** IoT network technologies for Hydrogen 4.0.

	Approx. Max.	Approx. Max.	Typ. Operating	Channel	Typ.	Relative Power	Relative
**Technology**	**Range** ^**1**^	**Data Rate** ^**1**^	**Frequency**	**Bandwidth**	**Latency**	**Consumption**	**Cost** ^**2**^
ZigBee	100 m	250 kbit/s	868 and 915 MHz ^3^, 2.4 GHz	2 MHz	30 ms	Low	Medium
Bluetooth	50 m	3 Mbit/s	2.4 GHz	1 MHz	100 ms	Medium	Low
BLE	10 m	1 Mbit/s	2.4 GHz	2 MHz	6 ms	Very Low	Low
LTE	10 km	12 Mbit/s	410 to 5925 MHz ^4^	1 to 20 MHz ^4^	50 ms	High	High
Wi-Fi	100 m	54 Mbit/s ^5^	2.4 and 5 GHz	20 to 80 MHz	50 ms	High	High
LoRa	5 m (urban), 15 m (rural)	50 kbit/s	169, 433, 868 and 915 MHz ^4^	125 kHz	100 to 1400 ms	Low	Low
Sigfox	10 m (urban), 40 m (rural)	100 bit/s	862 to 928 MHz ^4^	100 Hz	6 s	Low	Low
NB-IoT	10 m (urban), 50 m (rural)	200 kbit/s	461 to 2200 MHz ^4^	180 kHz	1.6 to 10 s	Medium	High

^1^ Theoretical maximum, highly dependent upon hardware selection and environmental conditions. ^2^ Inclusive of cost of equipment plus ongoing licensing or subscription costs. ^3^ The current revision of the ZigBee standard (3.0) allows use of sub-1 GHz bands but is unpopular due to country-specific band licensing. ^4^ Dependent upon hardware selection, region, and carrier. ^5^ For 802.11 g. Modern Wi-Fi standards enable data rates up to 9.6 Gbit/s (Wi-Fi 6); however. such speeds are of minimal advantage in IoT applications.

## Data Availability

The original contributions presented in the study are included in the article, further inquiries can be directed to the corresponding author.

## Abbreviations

The following abbreviations are used in this manuscript:

AWS	Amazon Web Services
BLE	Bluetooth Low-Energy
LTE	long-term evolution
IoT	Internet of Things
IIoT	Industrial Internet of Things
NB-IoT	Narrowband Internet of Things
HTTP	hypertext transfer protocol
TCP	transmission control protocol
UDP	user datagram protocol
MQTT	message queue telemetry transport
RSA	Rivest–Shamir–Adleman
ECC	elliptic curve cryptography
SCADA	supervisory control and data acquisition
IGCC	integrated gasification combined cycle
FCEV	fuel-cell electric vehicle
AI	artificial intelligence
ML	machine learning
RBAC	role-based access control
TLS	transport layer security
JSON	JavaScript object notation
HART	highway addressable remote transducer
redox	oxidation reduction
PV	photovoltaic
OC	open circuit
SC	short circuit
AC	alternating current
DC	direct current
MPP	maximum power point
MPPT	maximum power point tracker
SEPIC	single-ended primary-inductor converter
CCS	carbon capture and storage
SMR	steam methane reforming
POM	partial oxidation of methane
ATR	auto-thermal reforming
CG	coal gasification
SCG	surface coal gasification
UCG	underground coal gasification
PO	partial oxidation
AEL	alkaline electrolysis
CCM	catalyst-coated membrane
PEM	proton exchange membrane
AEM	anion exchange membrane
SOEL	solid-oxide electrolysis
AP	acidification potential
MOx	metal oxide
DT	digital twin
EC	electrochemical
TC	thermal conductivity
LEL	lower explosive limit
MEMS	micro-electromechanical systems
IRENA	International Renewable Energy Agency
H_2_	hydrogen
O_2_	oxygen
H_2_O	Water
CO	carbon monoxide
CO_2_	carbon dioxide
NH_3_	ammonia
C_7_H_8_	toluene
